# PPAR-Targeted Therapies in the Treatment of Non-Alcoholic Fatty Liver Disease in Diabetic Patients

**DOI:** 10.3390/ijms23084305

**Published:** 2022-04-13

**Authors:** Naomi F. Lange, Vanessa Graf, Cyrielle Caussy, Jean-François Dufour

**Affiliations:** 1 Department of Visceral Surgery and Medicine, Inselspital, Bern University Hospital, University of Bern, 3010 Bern, Switzerland; 2 Graduate School for Health Sciences, University of Bern, 3012 Bern, Switzerland; 3 Department of Diabetes, Endocrinology, Clinical Nutrition, and Metabolism, Inselspital, Bern University Hospital, University of Bern, 3010 Bern, Switzerland; vanessa.graf@insel.ch; 4 Univ Lyon, CarMen Laboratory, INSERM, INRA, INSA Lyon, Université Claude Bernard Lyon 1, 69495 Pierre-Bénite, France; cyrielle.caussy@chu-lyon.fr; 5 Département Endocrinologie, Diabète et Nutrition, Hôpital Lyon Sud, Hospices Civils de Lyon, 69495 Pierre-Bénite, France; 6 Centre des Maladies Digestives, 1003 Lausanne, Switzerland; 7 Swiss NASH Foundation, 3011 Bern, Switzerland

**Keywords:** non-alcoholic fatty liver disease (NAFLD), non-alcoholic steatohepatitis (NASH), type 2 diabetes mellitus, peroxisome proliferator-activated receptors (PPAR)

## Abstract

Peroxisome proliferator-activated receptors (PPAR), ligand-activated transcription factors of the nuclear hormone receptor superfamily, have been identified as key metabolic regulators in the liver, skeletal muscle, and adipose tissue, among others. As a leading cause of liver disease worldwide, non-alcoholic fatty liver disease (NAFLD) and non-alcoholic steatohepatitis (NASH) cause a significant burden worldwide and therapeutic strategies are needed. This review provides an overview of the evidence on PPAR-targeted treatment of NAFLD and NASH in individuals with type 2 diabetes mellitus. We considered current evidence from clinical trials and observational studies as well as the impact of treatment on comorbid metabolic conditions such as obesity, dyslipidemia, and cardiovascular disease. Future areas of research, such as possible sexually dimorphic effects of PPAR-targeted therapies, are briefly reviewed.

## 1. Introduction

As a leading cause of liver disease worldwide, non-alcoholic fatty liver disease (NAFLD) and non-alcoholic steatohepatitis (NASH) cause a significant burden [1]. NAFLD is a common comorbidity especially among individuals living with type 2 diabetes mellitus (T2DM) [2]. The complex bidirectional pathophysiological relationships between NAFLD and other metabolic diseases, particularly T2DM [3,4], demand a holistic and interdisciplinary approach to the treatment of NAFLD [5].

T2DM represents a major risk factor for NAFLD with over 55% of persons living with T2DM being affected by NAFLD [2]. T2DM furthermore predisposes individuals to advanced NAFLD, including development of NASH and liver fibrosis, and increases the risk of hepatocellular carcinoma [6,7]. NAFLD, in turn, increases the risk of incident T2DM [8]. Among NAFLD patients with advanced fibrosis, the majority have T2DM [9]. This complex population with multiple metabolic alterations such as NAFLD and T2DM should specifically be considered in the evaluation of potential pharmacological treatment strategies for NAFLD.

Peroxisome proliferator-activated receptors (PPAR), ligand-activated transcription factors of the nuclear hormone receptor superfamily, have been identified as key metabolic regulators in the liver, skeletal muscle, and adipose tissue, among others [10,11]. PPAR modulation has long been employed in the pharmacological treatment of multiple conditions, predominantly metabolic diseases such as T2DM and dyslipidemia, but has also been examined in the context of liver disease [12,13,14]. Considering the pathophysiological and epidemiological links between these conditions and NAFLD, PPAR modulators are being examined regarding their effects on NAFLD [15,16].

In the following, we will review the clinical evidence on PPAR-directed therapy for NAFLD, focusing on results and considerations in patients with type 2 diabetes mellitus.

## 2. PPAR Agonists in the Treatment of NAFLD with Concomitant T2DM

**Figure 1:**
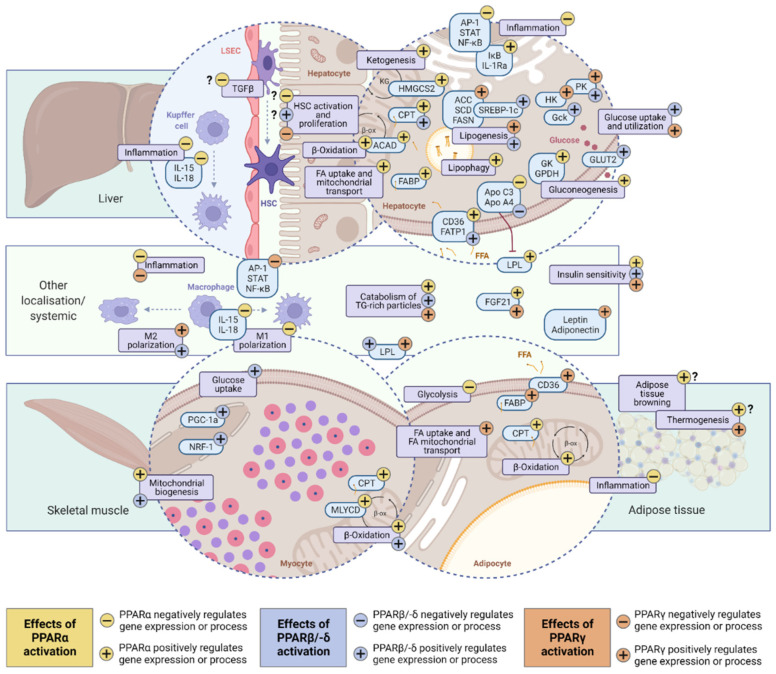
Overview of main tissue-specific and systemic effects of PPAR activation. Blue fields indicate proteins, purple fields indicate biochemical processes. Question marks indicate effects that are suspected but not confirmed. Red bars indicate inhibition. Created with BioRender.com. Abbreviations: β-ox, beta oxidation; ACAD, acyl-CoA dehydrogenases; ACC, acetyl-CoA carboxylase; AP-1, activator protein-1; Apo A4, apolipoprotein A4; Apo C3, apolipoprotein C3; CPT, carnitine palmitoyltransferases; FA, fatty acid; FABP, fatty acid binding protein; FASN, fatty acid synthase; FATP1, fatty acid transport protein-1; FFA, free fatty acid; FGF21, fibroblast growth factor 21; Gck, glucokinase; GK, glycerol kinase; GLUT2, glucose transporter 2; GPDH, glycerol 3-phosphate dehydrogenase; HK, hexokinase; HMGCS2, 3-hydroxy-3-methylglutaryl-CoA synthase 2; HSC, hepatic stellate cell; IκB, inhibitor of nuclear factor kappa B; KG, ketogenesis; IL-15, interleukin 15; IL-18, interleukin 18; IL-1Ra, interleukin-1 receptor antagonist; LPL, lipoprotein lipase; LSEC, liver sinusoidal endothelial cell; MLYCD, malonyl-CoA decarboxylase; NF-κB, nuclear factor kappa B; NRF-1, nuclear respiratory factor 1; PK, pyruvate kinase; PGC-1a, PPARG coactivator 1 alpha; SCD, stearoyl-CoA desaturase; SREBP-1c, sterol regulatory element binding protein 1; STAT, signal transducer and activator of transcription family; TG, triglyceride; TGFβ, transforming growth factor β.

The three PPAR isotypes, PPARα, PPARβ/PPARδ, and PPARγ in humans compose the 1C subfamily of the nuclear hormone receptor superfamily, which encompasses a large group of ligand-regulated transcription factors that share a common modular structure [17]. Tissue expression and effects of activation vary by isotype and tissue, demonstrating both redundant as well as distinct effects (reviewed in detail by [11,18]). Figure 1 provides an overview of tissue-specific and systemic PPAR main functions. Overall, PPAR isotypes exert pleiotropic functions in multiple tissues and pathways relating mainly to metabolism and immunity, which can induce reduction of hepatic steatosis and improvement of liver inflammation in patients with NASH [18]. Selected PPAR-agonistic molecules have demonstrated anti-fibrotic properties in the context of NAFLD [19,20]. As such, PPAR represents important targets in the treatment of NAFLD [15].

### 2.1. Molecular Basics of PPAR-Dependent Regulation

PPAR-dependent metabolic regulation of transcriptional activity occurs via several mechanisms. Firstly, ligand-dependent PPAR activation (ligand-dependent transactivation) prompts corepressor dissociation followed by heterodimerization with retinoid X receptors (RXR) and recruitment of a co-activator. The activated heterodimer proceeds to bind specific DNA sequences in the promotor regions of target genes, i.e., PPAR-responsive elements (PPREs) [10,21]. This PPRE-dependent mechanism leads to increased transcription of target genes. A multitude of both specific and shared ligands of PPARs has been identified, including natural as well as synthetic ligands [11].

PPAR may also regulate gene transcription negatively. Ligand-dependent transrepression describes a protein-protein interaction that leads to decreased transcription of predominantly inflammatory genes by interacting with transcription factors, such as members of the nuclear factor κB (NF-κB) family, and is independent of binding to a receptor-specific response element. Conversely, ligand-independent repression requires binding to PPRE, followed by recruitment of co-repressors. These mechanisms are comprehensively reviewed elsewhere [21]. Anti-inflammatory mechanisms of PPAR are mostly regulated through transrepression [18].

### 2.2. PPARα (NR1C1)

In 1990, the first isoform of PPAR was identified in humans and later classified as PPARα, which is encoded on the *PPARA* (*NR1C1*) gene [22]. This discovery was fueled by exploration of the pharmacological mechanisms of fibrates, which had been produced since the 1950s [12]. Multiple other synthetic and endogenous ligands for PPARα have since been characterized, including phospholipids and fatty acids and their derivatives, such as eicosanoids [11,23].

PPARα is a major regulator of cellular energy homeostasis and as such is expressed predominantly in oxidative tissues, such as the liver, adipose tissue, skeletal muscle, heart, and kidneys [11,24]. In the liver, the nuclear receptor is expressed mainly in hepatocytes but also non-parenchymal cells, namely stellate cells and liver sinusoidal endothelial cells [25].

While PPARα is active in both the fed and fasting state, it has a central role predominantly in the adaptive response to the latter [26,27]. Main functions include the transcriptional regulation of lipid catabolism by modulating expression of genes that mediate triglyceride hydrolysis, fatty acid transport, and β-oxidation in liver, skeletal muscle, and adipose tissue [23,28,29]. Additionally, PPARα regulates ketogenesis, which has been found to be severely impaired in the absence of PPARα [26,27].

Other functions of PPARα that are related to NAFLD include direct anti-inflammatory effects, which have been found to be independent of its metabolic functions in the liver [30]. Anti-fibrogenic effects of PPARα may be mediated through these anti-inflammatory effects as well as other mechanisms. Findings from pre-clinical mouse models of diet- and thioacetamide-induced fibrosing NASH suggest that PPARα agonism indirectly ameliorates liver fibrosis through modulation of hepatic stellate cell activation and related pro-fibrogenic pathways [31,32]. Interestingly, several findings indicate sexually dimorphic responses to PPARα activation, which warrants further exploration in the clinical context of NAFLD [33,34]. Diurnal cycling of nuclear receptor expression has been identified in several instances, notably including variable expression of PPARα [35]. A detailed review of PPARα functions can be found here [36].

### 2.3. PPARδ (PPARβ; NR1C2)

The isoform PPARδ (also: PPARβ), encoded on the *NR1C2* gene on chromosome 6, has previously been identified as a target for several metabolic conditions, including NAFLD [37,38], as receptor modulation was found to increase insulin sensitivity and improve lipid profile, while reducing obesity [39,40,41].

The receptor is expressed most abundantly in skeletal and cardiac muscle tissue, as well as in brown and white adipose tissue, macrophages, and the liver [11,17,24]. In the liver, the receptor further demonstrates a ubiquitous expression pattern, being present in hepatocytes, hepatic stellate cells, liver sinusoidal endothelial cells, and Kupffer cells [25]. Endogenous ligands of PPARδ include fatty acids and eicosanoids [42].

PPARδ exerts beneficial metabolic functions through maintaining oxidative capacity of skeletal muscle and mediating the adaptive response to exercise, enhancing mitochondrial biogenesis, fatty acid oxidation, and glucose utilization [41,43,44]. An increase in mitochondria and mitochondrial proteins in skeletal muscle is facilitated by PPARδ-mediated increase in peroxisome proliferator-activated receptor gamma coactivator 1-alpha (PGC-1a) concentrations and nuclear respiratory factor (NRF-1) expression [45]. PPARδ further has a critical role in the regulation of hepatic metabolism. In the pre-clinical setting, hepatic PPARδ overexpression led to increased liver glucose utilization and de novo lipogenesis while changing lipid profiles towards an increased ratio of monounsaturated to saturated fatty acids [46]. Despite lipid accumulation, PPARδ-overexpressing cells displayed less damage [46]. Overall, these findings indicate that PPARδ regulates hepatic glucose and fatty acid metabolism, thus playing a pivotal role in hepatic energy substrate homeostasis [46]. Further evidence suggests that liver-specific PPARδ activation also modulates energy substrate homeostasis in skeletal muscle towards fatty acid oxidation [47]. Hepatic PPARδ further regulates genes involved in lipoprotein metabolism, thus accounting for its beneficial effects on lipid profiles, as well as pathways related to inflammation and immunity, including promotion of anti-inflammatory macrophage polarization [48,49]. Recently, two detailed reviews have summarized the regulation of metabolism via PPARδ with a focus on NAFLD etiopathogenesis [37,38].

### 2.4. PPARγ (NR1C3)

PPARγ, which is encoded by *NR1C3* on chromosome 3, exerts its main metabolic effects in adipose tissue, being expressed in white and brown adipose tissue, as well as in macrophages [24]. Two isotypes of the PPARγ receptor, PPARγ 1 and PPARγ 2, have been identified [50]. Among these isoforms, PPARγ 1 demonstrates a broader expression pattern, while PPARγ 2 is predominantly expressed in adipose tissue [51].

Similar to other PPAR isoforms, endogenous ligands of PPARγ are fatty acids and eicosanoids [11]. Synthetic agonists of PPARγ include the anti-diabetic treatments rosiglitazone and pioglitazone, but also arachidonic acid metabolite anti-inflammatory drugs such as ibuprofen and indomethacin as well as the dual agonist saroglitazar [42,52].

PPARγ beneficially affects metabolism mainly by improving adipose tissue adipogenesis and adipose tissue fatty acid uptake and expenditure [52,53,54]. Adipose-tissue-specific PPARγ deletion in a pre-clinical model leads to severe lipoatrophy, highlighting the role of PPARγ in adipocyte development [55]. PPARγ deletion in adipose tissue and liver has furthermore been linked to insulin resistance [54,55,56]. Accordingly, PPARγ activation, for example with thiazolidinediones, displays insulin-sensitizing properties [52]. PPARγ activation has also been demonstrated to increase levels of adiponectin, an anti-atherogenic adipokine [48]. The receptor further possesses anti-inflammatory properties, acting via modulation of macrophage polarization and attenuation of the NF-κB pathway [49,57,58]. Regarding direct anti-fibrotic properties, PPARγ activity is linked to hepatic stellate cells displaying a quiescent phenotype and reduced hepatic stellate cell proliferation [59,60].

## 3. Pharmacologic PPAR-Targeted Therapies

**Figure 2:**
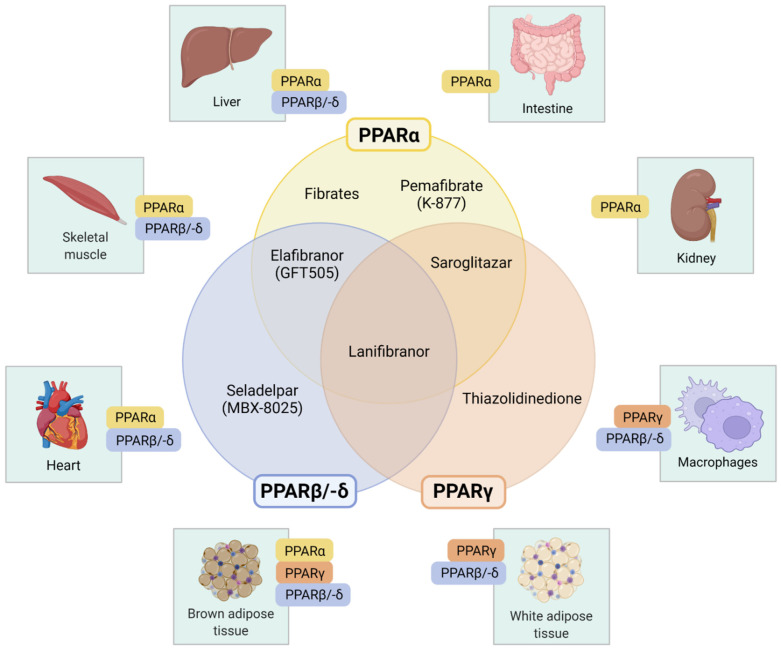
Overview of respective receptor profile of PPAR-modulating agents used in clinical trials and main tissue distribution of PPAR isotypes. Created with BioRender.com.

Several PPAR-modulating agents, with varying degrees of affinity for the different PPAR isotypes, have been investigated for the therapy of NAFLD and NASH (Figure 2). Table 1 provides an overview of recent controlled clinical trials reporting liver-related outcomes in patients with NAFLD.

### 3.1. Selective PPARα Modulator: Pemafibrate (K-877)

Pemafibrate is a selective PPARα modulator (SPPARMα), as among PPAR isotypes it is highly selective for PPARα [69]. Structural differences of the pemafibrate molecule compared to other PPARα agonists such as fenofibrate allow for this higher selectivity and agonistic activity at the receptor ligand-binding site [70]. The drug is currently approved and marketed in Japan for the treatment of dyslipidemia with high triglyceride (TG) and low high-density lipoprotein cholesterol (HDL-C) levels under the name Parmodia^®^ [71,72]. Thus, most evidence on pemafibrate in NAFLD is derived from trials conducted in this target population.

Pre-clinical data indicate a beneficial effect of pemafibrate on some aspects of liver histology in NAFLD/NASH. Steatosis, measured by area of oil red O staining but not hepatic TG content, inflammatory activity, and fibrosis improved under pemafibrate in a mouse model of diet-induced NASH [73]. In a STAM mouse model, mimicking NASH with underlying diabetes, pemafibrate ameliorated inflammatory activity while again no effect on hepatic TG content was observed [74].

A double-blind, randomized controlled phase 2 trial including 224 Japanese patients with dyslipidemia treated with pemafibrate twice daily (0.025 mg, n = 34; 0.05 mg, n = 37; 0.1 mg, n = 36; 0.2 mg, n = 36) versus fenofibrate once daily (100 mg, n = 36) or placebo (n = 34) over the course of 12 weeks has assessed the efficacy and safety of pemafibrate for the treatment of dyslipidemia [75]. The study population included 12% patients with T2DM and 20% with fatty liver. However, participants with poorly controlled T2DM (glycated hemoglobin A1c (HbA1c) ≥ 8.4%), history of hepatic impairment, and aspartate aminotransferase (AST) or alanine aminotransferase (ALT) levels more than 2-fold above the upper limit of normal (ULN) were excluded [75]. Pemafibrate showed a dose-dependent, significant reduction of plasma TG and increase in HDL-C levels compared to baseline and placebo, while elevations of AST and ALT occurred less frequently compared to fenofibrate [75].

Only patients with T2DM, 54% of whom had concomitant NAFLD (N = 166), were included in the randomized, placebo-controlled phase 3 PROVIDE trial (pemafibrate 0.2 mg/day, 0.4 mg/day, or placebo over 24 weeks), to assess the effect on fasting serum TG and further lipid-related as well as glycemic parameters [76]. The study demonstrated a significant decrease in TG levels by around 45% in both treatment groups. The treatment groups experienced fewer liver-related adverse events [76].

These findings were supported by a randomized controlled phase 3 trial, which included 223 Japanese patients with dyslipidemia, who received either pemafibrate 0.2 mg, 0.4 mg, or fenofibrate 106.6 mg daily [77]. Besides improvements in TG and HDL-C levels, the pemafibrate groups furthermore showed significant decreases in AST, ALT, gamma-glutamyltransferase (γ-GT), and alkaline phosphatase (ALP) [77]. However, only 5% of the participants had T2DM, no data on NAFLD is reported, and patients with poorly controlled T2DM or liver impairment were excluded [77].

Recently, a large post hoc analysis has summarized evidence from six, randomized controlled clinical trials of pemafibrate for treatment of dyslipidemia regarding outcomes related to glycemic control and liver values [62]. This pooled analysis included 1253 patients, 36% and 43% of whom had T2DM and fatty liver disease, respectively [62]. Among individuals with liver function tests above ULN at baseline, the proportion of patients with normalization of ALT, γ-GT, and ALP was significantly higher in the group treated with high-dose pemafibrate compared to placebo [62]. A significant decrease of liver tests was observed in all groups, predominantly in the 0.4 mg per day group [62]. Markers of glucose homeostasis, namely fasting plasma glucose and insulin, and homeostatic model assessment of insulin resistance (HOMA-IR) decreased significantly among the pemafibrate groups compared to placebo [62]. These data suggest a potential beneficial effect on both liver outcomes and glycemic control.

Few studies have assessed the effect of pemafibrate on liver-related outcomes other than blood-based markers. A single-arm, prospective trial investigated the efficacy and safety of pemafibrate 0.2 mg daily in patients with sonographically assessed NAFLD and dyslipidemia (N = 20, 40% T2DM) over the course of 12 weeks [78]. Elevated ALT levels decreased significantly in all participants (*p* = 0.001) and normalized in around half of the patients [78]. Furthermore, liver stiffness measurement (LSM) by vibration-controlled transient elastography (VCTE) and controlled attenuation parameter (CAP) decreased, but this did not reach statistical significance [78]. Similar findings were reported by a retrospective study that evaluated the effect of pemafibrate in 31 patients with NAFLD/NASH (16% T2DM), assessed non-invasively by FibroScan-aspartate aminotransferase (FAST) score, who also received pemafibrate 0.2 mg daily [79]. A significant decrease in FAST score over 48 weeks was observed, with a moderate, non-significant decrease in LSM [79].

Several other studies have retrospectively investigated pemafibrate in NAFLD and NASH [80,81,82]. Pemafibrate 0.2 mg was associated with improvements in several hepatic markers after a follow-up of one year in non-diabetic NAFLD patients [82] and in patients with biopsy-proven NASH [80].

To date, one randomized, placebo-controlled trial of pemafibrate 0.2 mg daily in patients with NAFLD (N = 118), defined as a liver fat content of ≥10% measured by magnetic-resonance-imaging-estimated proton density fat fraction (MRI-PDFF), has been reported [61]. In total, 36% of participants had T2DM and 67% had metabolic syndrome [61]. While no significant change in liver fat content was observed over 72 weeks, LSM by magnetic resonance elastography (MRE) significantly decreased by −6.2% (95% confidence interval [CI] −11.5 to −0.8, *p* = 0.024) in the pemafibrate group compared to placebo [61]. Among the six serious adverse events reported, none were related to pemafibrate [61].

Overall, the evidence regarding pemafibrate’s potential in the treatment of NAFLD patients with T2DM should be further explored. Currently only one randomized controlled trial has been reported and the number of patients with both NAFLD and T2DM in present studies is limited, hampering the extrapolation of findings to this population. Clinical trials evaluating the effect of pemafibrate on histological liver outcomes are currently missing.

### 3.2. PPARα Agonists: Fibrates

Fibrates were the first drug class utilizing PPARα agonism, albeit unknowingly, until discovery of the nuclear receptor [22]. Compared to pemafibrate, fibrates such as gemfibrozil, clofibrate, fenofibrate, and bezafibrate show relatively weak PPARα agonism [15,72]. While these substances are generally considered to be PPARα agonists, the individual receptor profile may differ between drugs. Bezafibrate, for instance, shows activity at all PPAR isotypes and may thus be classified as a pan-PPAR agonist [83]. Fibrates are currently used in the treatment of dyslipidemia and their potential to improve NAFLD has been explored for several decades [84]. In the field of liver disease, fibrates are further studied regarding use in primary biliary cholangitis (PBC) [85].

Among individuals with T2DM, serum levels of CC chemokine ligand 5 (CCL5) [86], a pro-inflammatory chemokine that has been implicated in advancing the development of fibrosis in NAFLD and NASH, decreased during fenofibrate treatment [87]. Recently, use of fibrates was identified as a protective factor against progression of NAFLD to advanced fibrosis (odds ratio [OR] 0.90, *p* < 0.05), defined as an increase in non-invasive markers, in a large cohort of American individuals with diabetes (N = 50,695) [7].

While promising results regarding hepatocellular damage and fibrosis have been reported in pre-clinical models [88,89], these findings have not translated into a clear benefit in clinical trials of NAFLD and NASH. In a small controlled clinical trial of gemfibrozil (N = 46), a decrease in transaminases, predominantly ALT, in NASH patients was noted [90]. In a prospective single-arm, dual biopsy trial of NAFLD patients (N = 16), only hepatocyte ballooning, but not other histological parameters, including steatosis, lobular inflammation, and fibrosis, changed significantly under fenofibrate (200 mg/day) over 48 weeks [91]. Treatment decreased TG, γ-GT, and ALP, but not transaminases [91]. Only one participant in this trial had T2DM [91].

Randomized, placebo-controlled trials have assessed the effect of fenofibrate in individuals without T2DM. Among 25 participants with insulin resistance and metabolic syndrome, fenofibrate reduced plasma TG as well as inflammatory markers interleukin-6 (IL-6) and high-sensitivity C-reactive protein (hsCRP), but did not improve insulin sensitivity [92]. Another trial assigned 27 patients with NAFLD (intrahepatic triglyceride (IHTG) content by magnetic resonance imaging ((MRI) ≥ 5.6%) and obesity to receive fenofibrate, niacin or placebo [93]. Fenofibrate decreased plasma TG and very low-density lipoprotein (VLDL) composition regarding TG and apolipoprotein B (apoB) content, while having no effect on insulin sensitivity or IHTG content [93]. Another clinical trial of non-diabetic NAFLD patients reported an increase in total liver volume and total liver fat volume under fenofibrate [94].

Thus, benefits of fibrates on liver-related outcomes in NAFLD currently seem limited, although data from clinical trials are largely based on individuals without T2DM. Previous trials failed to demonstrate insulin-sensitizing effects [92,93,95], but treatment might be warranted in certain dyslipidemic conditions (see Section 4.2.). Treatment seems safe regarding liver outcomes, as most commonly liver enzyme elevations are transient, although instances of acute liver injury during treatment with fibrates, mostly under fenofibrate, have been reported [96,97].

### 3.3. Selective PPARδ/β Agonist: Seladelpar (MBX-8025)

Seladelpar (MBX-8025) was developed as a selective PPARδ (PPARβ) agonist [98]. The molecule improved several parameters of glucose homeostasis and liver histology, including inflammation and steatosis, in a mouse model of NASH with T2DM and obesity [99]. In humans, seladelpar has previously demonstrated beneficial metabolic effects on atherogenic dyslipidemia (see Section 4.2), while no significant insulin-sensitizing effect was observed [98].

A randomized, placebo-controlled phase 2 trial (NCT03551522) of seladelpar in patients with histologically confirmed NASH provided preliminary results, demonstrating no effect on hepatic steatosis compared to placebo [18]. Final results, however, have not yet been published and development of the molecule has been halted for this indication after histological evaluation revealed findings of interface hepatitis [15].

### 3.4. PPARγ Agonists: Thiazolidinediones

Thiazolidinediones are a drug class of PPARγ agonists currently approved for the treatment of diabetes due to their insulin-sensitizing effects [52], with similar effects on glycemic control between different substances of the class [100]. Positive effects in NAFLD patients have been confirmed by several systematic reviews and meta-analyses [19,63,101,102]. A systematic review of eight randomized controlled trials of thiazolidinediones (pioglitazone and rosiglitazone) for treatment of NAFLD or NASH (15% T2DM), which was diagnosed based on histological criteria in the majority of cases, reported improvements in liver fat content and in serum levels of transaminases [63]. A meta-analysis of five clinical trials further indicated an improvement in lobular inflammation (risk ratio [RR] 1.72, 95% CI 1.33–2.22, *p*< 0.0001) and fibrosis (RR 1.39, 95% CI 1.01–1.90, *p* = 0.04) with thiazolidinedione (pioglitazone and rosiglitazone) treatment, although these latter findings did not consistently hold up in subgroup analyses [102]. Data obtained from another meta-analysis, which included eight randomized controlled clinical trials of patients with biopsy-confirmed NASH (overall N = 516 in analysis of primary outcome), indicated that only pioglitazone, but not rosiglitazone, leads to improvement of fibrosis ≥1 stage (OR 1.77, 95% CI 1.15–2.72, *p* = 0.009, and OR 1.18, 95% CI 0.43–3.25, *p* = 0.74, respectively) and NASH resolution (OR 2.14, 95% CI 0.94–4.86, *p*< 0.001, and OR 3.65, 95% CI 2.32–5.74, *p* = 0.07, respectively) [19].

#### 3.4.1. Pioglitazone

Pioglitazone also exhibits weak PPARα agonism, which may explain the beneficial effect on NAFLD compared with the other PPARγ agonists mentioned above [103]. Pioglitazone may currently be considered for treatment of NASH according to several international clinical guidelines [104,105,106]. Specifically, the clinical practice guidelines by the European Association for the Study of the Liver (EASL) and the American Association for the Study of the Liver (AASLD) state that treatment with pioglitazone may be discussed in patients with confirmed NASH with and without concomitant diabetes [107,108]. Recently, a network meta-analysis of 30 studies (N overall = 2356) found pioglitazone to be the most effective therapy for (NAS) reduction along with rosiglitazone and gastric bypass [109].

In a murine model of NAFLD, high-fat-diet-induced steatosis was ameliorated through pioglitazone administration by PPARγ- and PPARα-dependent increases of lipolysis, β-oxidation, and autophagy [110]. Improvement of hepatic steatosis by pioglitazone was found to be impaired in adiponectin knockout mice, indicating adiponectin involvement in the mechanisms exerted by pioglitazone [111]. Among human patients with T2DM, pioglitazone increased adiponectin levels, which correlated with improvements in parameters of glucose homeostasis [112].

Data on the anti-fibrotic effect of pioglitazone are not conclusive [19,102]. In different rat models of fibrosis (carbon tetrachloride, bile duct ligation, choline-deficient diet), the effect of pioglitazone treatment on fibrosis varied by the type of injury as well as stage of fibrosis at administration [113]. In humans, genetic factors have been implicated in the variation of response to pioglitazone [114] and in fibrosis regression among the Pioglitazone versus Vitamin E versus Placebo for the Treatment of Non-Diabetic Patients with Nonalcoholic Steatohepatitis (PIVENS) trial participants [115].

In the randomized, placebo-controlled PIVENS trial (N = 247), the effects of vitamin E and pioglitazone in NASH without T2DM were evaluated [116]. While significant reductions of transaminases compared to placebo were observed, the primary endpoint of a composite improvement in NASH histological features was not met in the pioglitazone 30 mg group after 96 weeks (34% vs. 19%, *p* = 0.04, pre-specified significance level of 0.025) [117]. While NAS improved significantly, fibrosis stage did not [117]. Histological resolution of steatohepatitis was found to be associated with fibrosis regression among participants of the PIVENS trial (OR 3.9, 95% CI 2.0 to 7.6, *p* < 0.001) [115].

The data suggest that a beneficial effect of pioglitazone is also present in T2DM patients and may even be more pronounced in this group compared to patients without T2DM. A placebo-controlled proof-of-concept study of 55 NASH patients with T2DM or prediabetes confirmed a beneficial effect on several metabolic and histological features, including steatosis and inflammatory activity [118]. Similarly, significantly more patients in the pioglitazone group reached the primary endpoint of a ≥ 2-point reduction in NAS compared to placebo (difference 41%, 95% CI 23% to 59%, *p* < 0.001) in a randomized controlled trial of patients with T2DM or prediabetes (N = 101) after 18 months [65]. In this trial, the mean change in fibrosis score was greater in the treatment group (difference −0.5, 95% CI −0.9 to 0) [65].

The data from this randomized placebo-controlled trial were evaluated specifically with regard to the effect of pioglitazone in T2DM compared to prediabetes [66]. NASH resolution under pioglitazone compared to placebo occurred significantly more often only in the T2DM patients (60% vs. 1%, *p* = 0.002), but not in patients with prediabetes (55% vs. 29%, *p* = 0.12), owing also to a high resolution rate under placebo in the prediabetes group [66]. Similarly, the improvement in fibrosis stage was significant only in the T2DM treatment group (−0.5 ± 0.9 vs. 0.2 ± 1.2, *p* = 0.042), but, as would be expected, this group also presented significantly higher baseline fibrosis scores [66]. An 18-month proof-of-concept study of the combination therapy of vitamin E 400 international units (IU) and pioglitazone 30–45 mg in patients with T2DM and bioptically confirmed NASH (N = 105) showed that a ≥ 2-point improvement in NAS (difference 35%, 95% CI 14–56%, *p* = 0.003) and NASH resolution (difference 31%, 95% CI 11–50%, *p* = 0.005) occurred more often in the combination than the placebo group [64]. The proportion of patients achieving an improvement in fibrosis stage did not differ significantly between groups (52% vs. 30%, *p* = 0.07) [64].

The effect of pioglitazone on fibrosis improvement in T2DM and prediabetes thus remains inconclusive. A meta-analysis of three of the above-mentioned trials along with one Chinese trial of pioglitazone versus berberine indicated significant improvements in steatosis, lobular inflammation, and ballooning, but not fibrosis [119].

Besides effects on NAFLD, pioglitazone is furthermore a strong insulin sensitizer and has a protective effect on beta-cell function, delaying the onset of T2DM in individuals with impaired glucose tolerance and impaired fasting glucose [120,121]. This was demonstrated in several clinical trials, for example the Actos Now for Prevention of Diabetes (ACT NOW) [122] and the Insulin Resistance Intervention After Stroke (IRIS) trials [123], which showed a 72% (*p* < 0.001) and 52% (*p* < 0.0001) risk reduction of development of overt T2DM under pioglitazone, respectively.

Despite strong indications that pioglitazone exerts multiple metabolic benefits in patients with T2DM, widespread use of pioglitazone is hampered by adverse effects. Besides weight gain (see Section 4.1), pioglitazone has been implicated in increasing the risk of bladder cancer [124,125,126] and fractures by decreasing bone mineral density [127]. However, evidence on the association of bladder cancer with pioglitazone remains inconclusive. One meta-analysis concluded that the risk of bladder cancer was not increased significantly with pioglitazone use (hazard ratio [HR] 1.07, 95% CI 0.96 to 1.18) [126]. A more recent systematic review and meta-analysis came to a similar conclusion when assessing data from randomized controlled clinical trials, but did find a significantly increased risk among subjects in observational studies (OR 1.13, 95% CI 1.03 to 1.25) [124]. Another systematic review of observational trials stated that existing data were too heterogeneous to derive a reliable conclusion [125]. An increased risk of bladder cancer was not described for rosiglitazone, indicating a possible adverse effect specific to pioglitazone rather than the drug class of thiazolidinediones [128].

Among individuals with NASH and T2DM/prediabetes, randomized to receive either pioglitazone 45 mg or placebo, use of pioglitazone was associated with a decrease of bone mineral density at the level of the lumbar spine at 18 months (−3.5%, *p* = 0.002) [127]. Bone mineral density did not change during the extension phase until 36 months and no low-energy fractures were reported [127]. Overall, pioglitazone might be a viable option for treatment of NASH in patients with T2DM, but individual risks and benefits should be carefully weighed. Several trials of pioglitazone in NAFLD treatment are currently ongoing (Table 2).

#### 3.4.2. Rosiglitazone

The thiazolidinedione rosiglitazone has been withdrawn from the European market and its use is restricted in the United States of America (USA) due to concerns regarding cardiovascular safety (see Section 4.3). With regard to NAFLD, positive results have previously been reported.

In a small, single-arm study of NASH patients (N = 30), 50% of whom had impaired glucose tolerance or T2DM, resolution of NASH was observed in 10 out of 22 participants (45%) with consecutive biopsies under rosiglitazone 8 mg daily after 48 weeks [129]. Serum ALT levels improved significantly [129]. Similarly, in a single-arm trial that included only T2DM-NAFLD patients (N = 68), rosiglitazone treatment over 24 weeks led to a reduction of liver enzymes and improvement in glycemic control [130].

The Fatty Liver Improvement with Rosiglitazone (FLIRT) randomized placebo-controlled trial was conducted in 63 patients with histologically confirmed NASH [131]. This trial showed significant steatosis improvement (≥30%) in 47% of participants under rosiglitazone versus 16% under placebo (*p* = 0.014), but failed to demonstrate a benefit regarding other histologic outcomes after one year [131]. Interestingly, absence of diabetes was identified as a predictor of treatment response in this trial [131]. Among 44 patients who completed an open-label, one-year extension phase of this trial, prolonged treatment with rosiglitazone did not improve fibrosis stage or inflammatory activity [132].

The effect of rosiglitazone was further explored in combination therapies. Rosiglitazone 8 mg daily alone or with either metformin or the angiotensin receptor blocker losartan was compared in a randomized, open-label trial of paired biopsies in patients with confirmed NASH (N = 135) [133]. This trial showed no difference in NASH histology, including steatosis, hepatocellular inflammation, and fibrosis, among the three treatment groups [133]. Contrary to rosiglitazone alone and in combination with losartan, no weight gain was observed in the group taking rosiglitazone in combination with metformin, but this difference failed to reach statistical significance, indicating that metformin did not sufficiently ameliorate weight gain under rosiglitazone [133].

More recently, an analysis of hepatic gene expression patterns in the treatment group of the FLIRT trial revealed increased expression of hepatic PPARγ and pro-inflammatory genes, indicating a potentially detrimental long-term effect of rosiglitazone treatment [134]. Given these findings and the concerns regarding cardiovascular adverse effects, rosiglitazone’s role in the treatment of NASH is currently limited.

#### 3.4.3. Lobeglitazone

Lobeglitazone is a more recently developed thiazolidinedione that along with PPARγ agonism also exerts partial PPARα-agonism, similarly to pioglitazone. Lobeglitazone is currently approved and marketed in Korea as an anti-diabetes drug under the name Duvie^®^ [42].

In a murine model of diet-induced NAFLD with obesity (high-fat diet), lobeglitazone administration for 4 weeks improved glucose homeostasis, hepatic steatosis, and serum lipid profile, accompanied by upregulation of hepatic gene expression related to fatty acid β-oxidation and decrease of genes involved in lipid synthesis and hepatic gluconeogenesis [135].

Data in human NAFLD is sparse. In a single-arm trial, 50 participants with T2DM and NAFLD, defined as controlled-attenuation parameter (CAP) over 250 dB/m, received lobeglitazone 0.5 mg for 24 weeks [136]. A modest but significant decline in hepatic steatosis, assessed non-invasively by CAP, compared to baseline was observed (313.4 dB/m vs. 297.8 dB/m, *p* = 0.016) [136]. Patients furthermore showed an improvement in glycemic control and atherogenic dyslipidemia [136].

### 3.5. Dual PPARα and -γ Agonist: Saroglitazar

Given the positive findings regarding dual agonism at PPARα and PPARγ with drugs such as pioglitazone, therapies specifically targeting both receptors for treatment of metabolic conditions were developed [137]. Recently one dual agonist, saroglitazar, was approved for NASH treatment in India (Lipaglyn^®^) [42]. The drug has previously been approved and marketed in India for treatment of diabetic dyslipidemia [138,139]. Compared to pioglitazone, saroglitazar exerts potent PPARα and only modest PPARγ agonism [140].

Data from pre-clinical, in vivo studies in rodent models indicate an improvement of insulin sensitivity, lipid profile, and other metabolic parameters with saroglitazar administration, while exhibiting a good safety profile [140]. In in vitro models of NASH (palmitic-acid-treated HepG2 and HepG2-LX2 co-cultures), saroglitazar showed a beneficial effect on several mechanisms involved in NASH pathogenesis [141]. In a mouse model of diet-induced NASH (high-fat, choline-deficient diet), saroglitazar demonstrated a more pronounced improvement in NAS compared to pioglitazone or fenofibrate [141]. Observed beneficial effects regarding fibrosis and fibrotic biomarkers were further confirmed in a mouse model of carbon tetrachloride (CCl_4_)-induced fibrosis [141]. These observations are in line with findings reported from a diet-induced mouse model of NASH induced by Western high-fat diet and sugar water [142]. In this model, saroglitazar improved all histologic features of NASH, including fibrosis stage, and led to resolution of NASH in the treatment group [142].

In clinical trials in participants with diabetes, saroglitazar has demonstrated beneficial effects on atherogenic dyslipidemia and insulin sensitivity. Decreases of TG, low-density lipoprotein cholesterol (LDL-C), and fasting plasma glucose were observed in a placebo-controlled trial of saroglitazar 2 mg or 4 mg in T2DM patients (N = 302) [143]. In a three-arm trial of individuals with T2DM (N = 122), higher-dose saroglitazar (4 mg) showed significant improvements in TG and LDL-C compared to pioglitazone [144]. No serious adverse events were observed under saroglitazar [144]. Insulin-sensitizing effects in T2DM were demonstrated more recently in a small randomized, placebo-controlled trial (N = 30) [145].

A systematic review evaluated the effect of saroglitazar in three clinical trials, currently published as abstracts, demonstrating liver-related outcomes in NAFLD patients with dyslipidemia [139]. Saroglitazar was shown to improve hepatic steatosis, assessed non-invasively by CAP, and plasma ALT levels [139]. Further evidence of saroglitazar in NAFLD exists from observational studies. In two prospective observational studies, patients with T2DM and NAFLD on ultrasound, who received saroglitazar 4 mg for 24 weeks, showed a significant improvement in transaminases, LSM, and steatosis, measured by CAP [146,147].

Promising findings from two phase 2 clinical trials of saroglitazar in NAFLD/NASH have recently been published [67,148]. A paired biopsy, controlled trial randomized 16 patients with histologically confirmed NASH to receive either saroglitazar 2 mg or 4 mg, or placebo over 24 weeks [148]. NAS decreased in both treatment groups (−1.5 ± 0.84, *p* = 0.77 in 2 mg; −1.9 ± 1.57, *p* = 0.60 in 4 mg), but differences were not statistically significant compared to placebo (−1.33 ± 0.58) [148]. NASH resolution without worsening of fibrosis occurred in three (4 mg) and four (2 mg) patients of the treatment groups compared to none under placebo [148]. In the four-arm, double-blind, randomized, controlled EVIDENCES IV trial (NCT0306172), 106 patients (52% T2DM) with obesity and NAFLD according to imaging or biopsy were randomized to receive saroglitazar (1 mg, 2 mg, or 4 mg) or placebo for 16 weeks [67]. Patients in all treatment arms achieved significant reductions in ALT, AST, ALP, and γ-GT [67]. Liver fat content on MRI-PDFF decreased significantly in the high saroglitazar dose compared to placebo (difference −23.8%, 95% CI −39.9 to −7.7, *p* = 0.004) [67]. Fibrosis markers decreased but did not differ significantly from placebo [67,148].

Saroglitazar has exhibited a favorable safety profile [139]. For other molecules of this drug class, adverse events were similar to pioglitazone, including edema and weight gain [149]. Data on the use of saroglitazar currently seem promising although evidence from larger trials with histological endpoints are lacking. Several phase 2 and 3 clinical trials are currently ongoing to evaluate the use of saroglitazar in NAFLD (Table 2).

### 3.6. Dual PPARα and -δ Agonist: Elafibranor (GFT505)

Elafibranor (GFT505) is a dual agonist of PPARα and PPARδ, with predominant activity on the former [150]. In several rodent models of NAFLD/NASH, elafibranor administration decreased expression of pro-fibrotic and pro-inflammatory genes and improved various histological outcomes, including steatosis, inflammation, and fibrosis [151]. In an in vitro model of NASH, elafibranor was found to exert the strongest anti-NASH effects compared to seven other PPAR-modulating agents [152].

In humans, elafibranor enhanced insulin sensitivity in liver and muscle tissue, and reduced plasma TG, LDL-C, and ALT levels [153]. In the phase 2, randomized controlled GOLDEN-505 study of 274 patients with histologically confirmed NASH (39% T2DM), individuals treated with elafibranor 120 mg over 52 weeks had higher rates of NASH resolution without worsening of fibrosis compared to placebo (19% vs. 12%, OR 2.31, 95% CI 1.02 to 5.24, *p* = 0.045) [68]. The effect was more pronounced in individuals with NAS of ≥4 at baseline (OR 3.52, 95% CI 1.32 to 9.40, *p* = 0.013) [68]. Importantly, these analyses were performed according to the revised definition of treatment response while the protocol-defined primary endpoint was not met [68].

Elafibranor subsequently went on to the phase 3 RESOLVE-IT trial (NCT02704403), but the development has been halted after an interim analysis failed to achieve the primary endpoint of NASH resolution without worsening of fibrosis [154,155].

### 3.7. Pan-PPAR-Agonist: Lanifibranor (IVA337)

Lanifibranor is an indole sulfonamide derivative and a balanced pan-PPAR agonist that has demonstrated strong therapeutic potential in pre-clinical models of NAFLD/NASH [156]. Specifically, lanifibranor ameliorated insulin resistance and improved histological features of NASH, including steatosis, ballooning, and inflammation, in diet-induced and genetic animal models [157]. Lanifibranor showed both therapeutic as well as preventive anti-fibrotic properties in a CCl_4_-induced model of fibrosis, inhibiting the expression of pro-fibrotic and inflammatory genes [157]. In a mouse model of diet-induced NASH, the ameliorative effects of lanifibranor on certain aspects of NASH histology were greater than those observed with agonists of individual PPARs [158]. While macrophage infiltration due to acute CCl_4_-induced injury remained unchanged under lanifibranor, macrophages displayed a metabolically activated phenotype, decreasing inflammation [158]. In vivo and in vitro models further indicate a beneficial effect on portal hypertension. In rat models of cirrhotic liver disease (bile duct ligation, thioacetamide exposure), lanifibranor lowered portal pressure, improved microvascular function, and attenuated fibrosis [159]. These findings indicate potential in the treatment of advanced chronic liver disease.

The impact of lanifibranor in human NASH has been evaluated in the randomized, placebo-controlled phase 2 NATIVE trial (NCT03008070) [160]. In total, 247 patients with non-cirrhotic (fibrosis stages F2–3), histologically active NASH (42% T2DM) were randomized to receive either lanifibranor (1200 mg or 800 mg) or placebo over 24 weeks [20]. The primary endpoint of decrease in histological activity (≥2 points in the activity score SAF-A) without worsening of fibrosis was significantly more likely in the higher dosage treatment group compared to placebo (55% vs. 33%, RR 1.69, 95% CI 1.22 to 2.34, *p* = 0.007), while no significant improvement was observed with the lower dose (48% vs. 33%, RR 1.45, 95% CI 1.00 to 2.10 *p* = 0.07) [20]. An improvement of ≥1 fibrosis stage without worsening of NASH also occurred more often in the high-dose lanifibranor group compared to placebo (RR 1.68, 95% CI 1.15 to 2.46) [20]. A network meta-analysis of pharmacologic therapies for NAFLD ranked the probability of achieving an improvement of ≥1 fibrosis stage as being highest with lanifibranor (OR 2.38, 95% CI 1.21 to 4.67) [161]. Lanifibranor is one of the two pharmacological therapies that have demonstrated an improvement in fibrosis stage in clinical trials. The efficacy of lanifibranor is currently investigated in a phase 3 clinical trial in patients with NASH (NATiV3; NCT0484972).

## 4. Comorbidities of the Metabolic Syndrome in the PPAR-Targeted Treatment of Diabetic NAFLD Patients

Care of individuals with T2DM must consider co-existing conditions [162], and the presence of NAFLD or NASH adds further complexity to this population with multiple metabolic comorbidities [5]. The following chapter provides an overview of common comorbid conditions of the metabolic syndrome in T2DM patients and the possible impact of PPAR-directed therapies on these conditions. Findings are summarized in Table 3. Given the large volume of evidence published on these topics, we focused on pivotal trials and recent works summarizing previous findings.

### 4.1. Overweight and Obesity

Both NAFLD and T2DM are closely linked with obesity. The prevalence of obesity has been estimated at 51% among NAFLD patients, rising to 82% in patients with NASH [1]. Thus, pharmacological treatments for NAFLD should be evaluated with regard to their effects on weight, especially in diabetic patients.

Among the discussed PPAR-directed therapies, weight gain has consistently been reported for thiazolidinediones [100]. However, conflicting data exist as to whether this weight gain is predominantly associated with fluid retention or an increase in adipose tissue mass [163,164,165]. Possible cardiac implications of fluid retention are discussed in chapter 4.3. Recent data from obese women treated with pioglitazone 30 mg over 16 weeks compared to placebo indicate an increase in adipogenesis in the subcutaneous femoral adipose tissue depot, which is considered beneficial for metabolic health compared to other depots [166], while reducing visceral adipose tissue [167]. These findings are in line with other evidence demonstrating improved adipose tissue metabolism [165] and an overall beneficial cardiovascular effect of pioglitazone (see Section 4.3).

In the three-arm PIVENS trial of pioglitazone or vitamin E versus placebo, only the pioglitazone group demonstrated a significant weight gain of 4.7 kg (*p* < 0.001) [117]. Overall, trials have consistently reported a considerable increase of around 3–7% of body weight during thiazolidinedione treatment [117,118,129,168,169], which was also confirmed in participants with T2DM and prediabetes [65]. In a study by Bril et al. (2019), individuals with T2DM, who received combination therapy with vitamin E and pioglitazone, demonstrated a significant weight gain (5.7 ± 5.4 kg, *p* < 0.001) after 18 months compared to no significant changes in the vitamin E and placebo groups [64]. Weight gain was not ameliorated by combining pioglitazone with instructions regarding a hypocaloric diet [65,118]. Weight gain among NASH patients in a 48-week trial of pioglitazone partially remained at the 6-month post-treatment follow-up [129].

Inconsistent findings regarding weight gain have been reported from trials of dual or pan-PPAR agonists, which exert PPARγ agonism. Both bezafibrate and saroglitazar have demonstrated no effect on body weight [139,170]. In the context of bezafibrate, it has been discussed that this might be due to concomitant PPARδ activation, ameliorating PPARγ-mediated weight gain [170]. In contrast, weight gain has been observed for lanifibranor, the pan-PPAR agonist currently under investigation in NAFLD [20]. Francque et al. (2021) reported a 3% increase in body weight in both the low- and high-dose treatment group of the phase 2 NATIVE trial [20].

No clinically relevant changes in body weight have been reported for seladelpar [99], elafibranor [68], and fibrates, including fenofibrate [91] and pemafibrate [61].

### 4.2. Dyslipidemia

PPAR agonists have demonstrated effects mostly in the treatment of atherogenic dyslipidemia, which is a common comorbidity in T2DM patients [16,171]. Atherogenic dyslipidemia is defined by low plasma levels of HDL-C with elevated levels of TG and small and dense LDL-C [172]. Atherogenic dyslipidemia represents a major risk factor for cardiovascular disease. Effects of PPAR-agonists on cardiovascular outcomes are discussed in Section 4.3.

As fibrates reduce TG and, to a lesser extent, improve levels of HDL-C [36], the use of fibrates to reduce residual cardiovascular risk in persistent atherogenic dyslipidemia despite lifestyle or statin treatment in patients with T2DM has been evaluated [173]. The Fenofibrate Intervention and Event Lowering in Diabetes (FIELD) trial included 9795 T2DM patients without lipid-lowering treatment at baseline and without clear indication for the former [174]. At 2 years, TG levels in the fenofibrate treatment group compared to placebo were 21% and 29% lower in the subgroups of patients with and without other lipid-lowering treatment during the study period, respectively [174]. In the large randomized, controlled Action to Control Cardiovascular Risk in Diabetes (ACCORD) trial, T2DM patients with dyslipidemia (N = 5518) were treated with fenofibrate or placebo along with open-label simvastatin [175]. A mild increase of HDL-C, paralleling that in the placebo group, and regression in TG were observed in the fenofibrate group [175].

The selective PPARα agonist pemafibrate is currently approved in Japan for the treatment of hyperlipidemia [72]. A thorough, detailed review of the role of pemafibrate in the treatment of atherogenic dyslipidemia can be found here [176]. In the placebo-controlled, phase 3 PROVIDE trial, the use of pemafibrate led to a significant decrease of fasting TG compared to placebo (*p* < 0.001) [76], an effect that was stable during the open-label extension period [177]. A pooled analysis of six placebo-controlled phase 2 and 3 trials in a large cohort of 1253 patients further confirmed these findings in combination therapy [178]. After 12 weeks, TG levels in both statin users and non-users significantly declined by 45–50%, in a dose-dependent manner with pemafibrate doses ranging from 0.1 mg/day to 0.4 mg/day, while no significant changes were observed in the placebo groups (*p* < 0.001 vs. placebo) [178]. Currently available data indicate that the lipid-lowering effects of pemafibrate are comparable or superior to those of fibrates [77,179].

Lipid-modulating effects of pioglitazone were observed in the Pioglitazone Effect on Regression of Intravascular Sonographic Coronary Obstruction Prospective Evaluation (PERISCOPE) [180] and Carotid Intima-Media Thickness in Atherosclerosis Using Pioglitazone (CHICAGO) [181] trials. In the PERISCOPE trial, HDL-C levels significantly increased and TG levels decreased in 543 T2DM patients who received pioglitazone 15–45 mg/day versus glimepiride 1–4 mg/day [180]. This was accompanied by a reduction of coronary atherosclerosis progression with pioglitazone as measured by a decrease in percent atheroma volume in intravascular ultrasound [180]. A post hoc analysis revealed that atheroma regression was associated with changes in lipid levels [182]. In the CHICAGO trial, pioglitazone compared to glimepiride reduced carotid intima artery intima-media thickness in 462 patients with T2DM, which was found to be associated with improvements in HDL-C in a post hoc analysis [181,183].

Beneficial effects of saroglitazar regarding TG levels in T2DM patients have been reported in both randomized controlled trials as well as observational cohorts [184,185]. Saroglitazar is currently approved in India for treatment of atherogenic dyslipidemia in T2DM [185]. Recently, the randomized, controlled phase 3 PRESS XII trial evaluated the effect of saroglitazar 2 mg or 4 mg compared to pioglitazone on glycemic control and lipid profiles in 1155 patients with T2DM over 56 weeks [186]. Similarly, to participants in the pioglitazone arm, participants in the saroglitazar groups experienced a significant reduction of TG and LDL-C while HDL-C increased [186]. Information on use of other lipid-lowering agents, however, is not reported [186]. A recent meta-analysis of five randomized controlled trials confirms a benefit regarding TG reduction with saroglitazar compared to placebo or pioglitazone, but not compared to active control with other lipid-lowering agents (atorvastatin or fenofibrate) after 12 weeks [185]. Changes in HbA1c, LDL-C, or HDL-C levels were not significant [185].

Improvements in lipid profiles have also been demonstrated in the now discontinued agents seladelpar and elafibranor. In a randomized, placebo-controlled trial, seladelpar with or without atorvastatin significantly lowered LDL-C and TG, and increased HDL-C in individuals (N = 183) with dyslipidemia and abdominal obesity [98]. An improvement of lipoprotein subfractions was observed in another randomized, placebo-controlled trial with seladelpar alone or in combination with atorvastatin [187]. Compared to placebo, elafibranor significantly reduced fasting TG and increased HDL-C in a randomized, placebo-controlled trial of 141 patients with prediabetes or dyslipidemia, while LDL-lowering effects were only observed in the prediabetes group [188]. Similar findings were reported in the GOLDEN-505 trial in NASH patients [68].

Dyslipidemia is highly prevalent in individuals with NAFLD and T2DM, most of whom benefit from statin therapy, given the pleiotropic beneficial cardiovascular [189] as well as liver-related [190,191] effects of statins. Both FIELD and ACCORD trials showed overall low rates of myopathy under fenofibrate alone and under combination of fenofibrate with statins [174,175]. Overall, incidence rates of rhabdomyolysis in combination therapy were found to be lowest for fenofibrate combinations, although risk was higher in older and T2DM patients [192]. Among fibrates, gemfibrozil is associated with a higher risk of muscle-related adverse events in combination therapy with statins due to different pharmacokinetics, resulting in impaired statin metabolism [193]. Currently, statin-fibrate combination therapy may be considered in select patients with severe or refractory mixed dyslipidemia, intact renal function, and careful clinical follow-up [42].

In this context, the development of newer PPARα agonists for treatment of NASH further leads one to question the safety of these treatments, especially in combination with statins. Saroglitazar is specifically marketed for treatment of residual atherogenic dyslipidemia under statin treatment and has demonstrated a favorable safety profile regarding myopathy in the phase 3 PRESS VI trial, where it was combined with atorvastatin 10 mg [144]. Likewise, the SPPARMα pemafibrate demonstrated a good safety profile, regardless of statin use and mild renal dysfunction, in a pooled analysis of several randomized trials [178]. Effects of the Pan-PPAR agonist lanifibranor on lipid profiles were overall modest with no muscle-related adverse events in the phase 2 NATIVE trial [20].

### 4.3. Cardiovascular Comorbidities

T2DM, along with comorbid obesity and dyslipidemia, constitutes a major risk factor for cardiovascular disease (CVD) [194]. Moreover, several studies have provided evidence that NAFLD could be an independent CVD risk factor with a potential synergistic increased risk in patients with NAFLD and T2DM [195]. NAFLD patients are at risk of excess mortality from CVD, with the risk increasing with more advanced disease [196]. Treatment strategies in NAFLD should thus be considered with regard to their effect on cardiovascular conditions [197]. As PPAR modulation improves metabolism as well as endothelial dysfunction and inflammation [198], several PPAR-targeted therapies have been assessed regarding their potential to ameliorate cardiovascular disease and prevent cardiovascular events (reviewed in [23,42]).

Given their role in atherogenic dyslipidemia and their long-standing market approval, a lot of evidence exists regarding the effects of fibrates on cardiovascular outcomes [23]. The previously mentioned FIELD and ACCORD trials are two landmark studies of fibrates in the prevention of cardiovascular events in T2DM patients [174,175]. The randomized, controlled FIELD trial (N = 9795) included individuals with T2DM both with and without previous cardiovascular disease (approximately 1:4) and without specific indication for dyslipidemia treatment or presence of NAFLD [174]. While fenofibrate did not reduce the risk of major coronary events, it did reduce the incidence of non-fatal myocardial infarction and microvascular-associated complications [174]. Furthermore, events were significantly reduced in a subgroup analysis of those with dyslipidemia [174]. However, the ACCORD trial failed to demonstrate a reduction of the CVD risk compared to statin therapy alone in patients with T2DM at high risk for CVD [175]. In a meta-analysis of six primary prevention trials, including ACCORD and FIELD, it was determined that fibrates lower the risk of cardiovascular events (coronary heart disease death or non-fatal myocardial infarction) in primary prevention, although the absolute effect was rather modest with an absolute risk reduction of merely <1% [97]. The majority of patients included in the overall cohort had T2DM [97].

Regarding secondary prevention, a systematic review and meta-analysis concluded that fibrates were effective in the prevention of the composite outcome of non-fatal stroke, non-fatal myocardial infarction, and vascular death [199]. This analysis, however, included data on the drug clofibrate, which has been withdrawn from the market [199]. Whether these findings can be extrapolated to currently available fibrates is unclear [199].

As described above, the SPPARMα pemafibrate has demonstrated beneficial effects on atherogenic dyslipidemia. The effects of pemafibrate on reduction of cardiovascular events in diabetic patients are currently being investigated in the clinical Pemafibrate to Reduce Cardiovascular Outcomes by Reducing Triglycerides (PROMINENT) trial, which plans to enroll 10,000 subjects in 24 countries [200].

Another class of drugs that has been extensively studied for potential cardiovascular outcomes is thiazolidinediones, especially pioglitazone [120]. Pioglitazone has been demonstrated to improve certain parameters of cardiac metabolism and function in T2DM subjects, including myocardial insulin sensitivity, left ventricular diastolic function, and systolic function [201,202]. In the PERISCOPE trial of patients with coronary artery disease and T2DM, pioglitazone furthermore slowed the progression of coronary atherosclerotic lesions, assessed by intravascular ultrasound [180].

In the phase 3 PROspective pioglitAzone Clinical Trial In macroVascular Events (PROactive) trial, the use of pioglitazone in the high-risk group of diabetic patients with prior evidence of macrovascular disease was assessed [203]. After a mean follow-up of almost 3 years, pioglitazone failed to significantly improve the composite primary outcome, which included lower extremity revascularization among other cardiovascular endpoints such as mortality and myocardial infarction (HR 0.90, 95% CI 0.80 to 1.02, *p* = 0.095) [203]. Regarding the narrower secondary composite outcome of all-cause mortality, non-fatal myocardial infarction, and stroke, however, pioglitazone was superior to placebo (HR 0.84, 95% CI 0.72 to 0.98, *p* = 0.027) [203]. An individual patient data meta-analysis of 16,390 T2DM patients from 19 trials, including the PROactive trial, further confirmed this observation of risk reduction in the composite endpoint of mortality, myocardial infarction, and stroke (HR 0.82; 95% CI 0.72 to 0.94; *p* = 0.005) [204]. In the randomized, placebo-controlled Insulin Resistance Intervention After Stroke (IRIS) trial, pioglitazone has been shown to reduce the risk of stroke and myocardial infarction after a previous recent cerebrovascular event in patients with insulin resistance but without diabetes (HR 0.76; 95% CI 0.62 to 0.93; *p* = 0.007) [123,169]. In patients with prediabetes and good adherence to pioglitazone treatment (≥80%), the risk for acute coronary syndrome was reduced by 53% (95% CI 74% to 15%; *p* = 0.01) [205].

However, subjects receiving pioglitazone have also been found to be more likely to develop edema and difficulty breathing in the IRIS study [169]. PPARγ prompts fluid retention by increasing sodium avidity in the renal collecting ducts [206]. Data further indicate that fluid retention is a class effect of thiazolidinediones rather than an effect of individual drugs [207]. Among NASH patients, however, data suggest that weight gain may be attributable to an increase in adipose tissue rather than fluid retention, possibly indicating that this adverse effect might be less pronounced in this patient group [163].

As sodium and fluid retention exert deleterious effects on the cardiovascular system, the relationship between thiazolidinediones and heart failure has long been a matter of debate. In a large individual patient data meta-analysis of Lincoff et al. (2007), subjects in the pioglitazone group experienced serious heart failure significantly more often (HR 1.41; 95% CI 1.14 to 1.76; *p* = 0.002) [204]. However, this did not translate into an increased risk of overall mortality (HR 0.92; 95% CI 0.76 to 1.11; *p* = 0.38) [204]. A secondary analysis of the IRIS trial concluded that the risk of heart failure was not increased in individuals with non-diabetic insulin resistance after cerebrovascular events under pioglitazone compared to placebo (4.1% vs. 4.2%) [208]. Among patients with prediabetes and good adherence (≥ 80%), the risk of the composite endpoint stroke, myocardial infarction, and hospitalization for heart failure was reduced (HR 0.61; 95% CI 0.42 to 0.88; *p* = 0.008) despite a significantly higher rate of edema (37% vs. 25%; *p* < 0.001) [205]. In the IRIS trial, patients with pre-existing heart failure were excluded, participants were closely monitored by their providers, and dosage adjustments were performed where necessary, indicating that this complication of pioglitazone treatment may be managed clinically without increased risk of a negative outcome [208]. It has been hypothesized that weight gain may lead to overt heart failure only in patients with underlying, sub-clinical cardiac dysfunction rather than development of heart failure [197]. This seems plausible, given the high baseline prevalence of cardiac dysfunction in the group of patients with T2DM [209].

A controversy regarding increased risk of cardiovascular mortality with rosiglitazone has long been ongoing. A meta-analysis of 42 trials revealed a significantly increased odds ratio of 1.43 (95% CI 1.03 to 1.98; *p* = 0.03) for myocardial infarction as well as an increased, albeit not significant, odds ratio for death from cardiovascular causes 1.64 (95% CI 0.98 to 2.74, *p* = 0.06) [210]. Updated meta-analyses have supported these findings [211,212]. In contrast, a large open-label randomized controlled trial (N = 4447) of patients with type 2 diabetes, who received either rosiglitazone or a combination therapy with metformin and sulphonylureas, showed non-inferiority of rosiglitazone compared to the active control regarding the composite primary endpoint of cardiovascular hospitalization or cardiovascular death (HR 0.99, 95% CI 0.85 to 1.16) [213]. While the American FDA has lifted restrictions on the use of rosiglitazone, the approval of rosiglitazone by the EMA ended in 2010 [18].

As detailed below, other PPAR-modulating agents have demonstrated favorable effects on lipid profiles in diabetic patients, thus indicating possible beneficial effects in cardiovascular disease (see Section 3.3), although long-term cardiovascular safety has not been established. Notably, the partial PPARγ agonist saroglitazar has demonstrated a satisfactory cardiovascular safety profile in the short term [186]. Likewise, no cardiovascular safety concerns were raised for elafibranor [68].

In the context of cardiovascular conditions, it is worth noting that the antihypertensive agent telmisartan, an angiotensin receptor blocker, further exerts agonistic effects at PPARγ and -α. The role of telmisartan in the treatment of NAFLD has thus been evaluated for both its renin-angiotensin-system (RAS)- and PPAR-modulating properties [214]. In T2DM human subjects with arterial hypertension, telmisartan attenuated liver-spleen ratio, indicating an improvement in hepatic steatosis [215]. In transcriptome analyses, telmisartan was shown to ameliorate development of NASH in a mouse model of diabetic NASH (STAM) [216]. Further studies are needed to determine the effect of telmisartan in patients with NASH and liver fibrosis.

## 5. Outlook and Further Areas of Research

As outlined in the previous chapters, PPAR modulates a wide range of metabolic functions and elicits pleiotropic effects in multiple tissues. Adding further complexity, specific effects can be elicited and combined by the use of molecules with distinct activity profiles on multiple PPAR isotypes [11]. This presents major challenges for research into PPAR therapies for NAFLD, but also offers considerable opportunities. One aspect of NAFLD therapy that has elicited attention is the prospect of possible combination therapies, simultaneously acting on several targets and thus offering synergistic treatment effects [217]. Combination of PPAR-targeted therapies with other pharmacological agents in the treatment of NAFLD will warrant careful exploration, given the multi-systemic effects of PPAR modulation [217]. This holds true also for concomitant treatments targeted towards other components of the metabolic syndrome such as the combination of statin and fibrate therapy for dyslipidemia.

Similarly, an aspect of PPAR-targeted therapy needing further investigation is the interplay of pharmacologic agents with PPAR modulation derived from the individuals’ environment. Among the identified ligands of PPARs are so-called endocrine-disrupting chemicals (EDCs), which are defined as exogenous chemicals or mixtures of chemicals that interfere with any aspect of hormone action [218]. EDCs have been demonstrated to deregulate the activity of nuclear hormone receptors, such as PPAR isotypes and their heterodimerization partner RXR [219]. Subsequently, modulation of PPARs by EDCs has been discussed in the etiopathogenesis of NAFLD [220], obesity [221], and T2DM [222]. Although this may be far-reaching from a clinical point of view, the interaction between PPAR-modulating pharmacological agents and EDCs in NAFLD patients with metabolic conditions presents an interesting aspect for future research, especially given the high worldwide prevalence of NAFLD.

Other PPAR-directed environmental factors may be immediately influenced by lifestyle adjustments. As mentioned previously, nutrition-derived fatty acids and their metabolites have been identified as ligands for all PPAR isotypes [11]. As a key regulator of energy source homeostasis, PPARα, for example, mediates the response to acute fasting, while its involvement in the adaptive response to intermittent fasting is not fully elucidated [26,223,224]. PPAR activity may thus be directly or indirectly influenced by adjustments in nutrition and dietary patterns, especially fasting, as well as modulation of the gut microbiome [225,226]. Combination treatments of lifestyle interventions with pharmacologic PPAR-targeted therapy thus present an interesting area for future research. Ideally, clinical trials should consider these lifestyle-related factors to further elucidate possible synergistic mechanisms with PPAR-targeted therapies.

Closely related to their function as key regulators in metabolism and energy homeostasis is the diurnal cycling of several PPAR isotypes. Specifically, PPARα and PPARδ demonstrate diurnal expression and activity patterns, related to feeding status [35,47,227]. Circadian rhythm, encompassing the diurnal activity of several nuclear receptors, plays a pivotal role in metabolic homeostasis and disturbances of the former have been linked to NAFLD development [228]. Differences in the activation patterns of these receptors, as would be prompted by pharmacologic therapies, might elicit metabolic responses different to those observed with the natural fluctuation of PPAR activity. The extent to which this affects overall metabolism, circadian rhythm, and treatment effects will warrant further exploration [228].

Another aspect of research that has recently gathered interest is the sexual dimorphism of several metabolic conditions, including amongst others NAFLD and T2DM [229,230,231,232,233], although previous research in the field of NAFLD has often neglected to take these sex differences into account [234,235]. While the biological and social factors contributing to sex differences in metabolic and cardiovascular conditions as well as liver metabolism are complex and manifold (as reviewed here [236] and here [237]), one particular target of NAFLD treatment that has been identified as eliciting sexually dimorphic responses is PPARα [11]. In previous research, PPARα SUMOylation in females has been described as protecting the liver from estrogen-mediated intrahepatic cholestasis of pregnancy [34]. Recently, sexually dimorphic responses to PPARα activation by pemafibrate have been described in a rodent model [33]. Four models of diet-induced NAFLD elicited distinctly different responses in male compared to female mice, with transcriptome analysis indicating marked differences in genes regulated by PPARα [33]. Sexually dimorphic gene expression related to PPARα was subsequently demonstrated in human liver tissue samples from patients with NAFLD [33].

Because PPARα signaling is involved in the response to fasting, it could be hypothesized that differential responses to fasting in male and female rodents [238] as well as to dietary interventions in humans [239] might be mediated to some extent by PPARα. However, the degree to which these differences are conferred by sexual dimorphism in PPARα activation remains to be elucidated further, as numerous other factors and mechanisms, including estrogen signaling, strongly influence sex differences in the response to feeding and fasting [240,241]. Interestingly, several clinical trials of PPARα agonists in humans support a possible sexually dimorphic effect, although the results are inconclusive and the magnitude as well as the direction of the effect remains unclear. A subgroup analysis of the previously described ACCORD trial revealed a possible differential treatment effect, with sex showing a significant interaction with treatment, resulting in more favorable effects in men [175]. While improvement in lipid profiles was more pronounced in females in the FIELD trial, this did not translate into a significant difference regarding cardiovascular outcomes or significant interaction with treatment effect [174,242].

Data regarding possible sex differences in the other PPAR isoforms PPARδ and PPARγ are scarce. While glucose homeostasis and insulin sensitivity have been described as sexually dimorphic factors [243], the role of PPARγ as a main regulator of these processes in the context of these sex-specific findings is not well-described. Previous research clearly indicates sex hormone signaling as a central mediator of these processes [243], with PPARγ interacting with these pathways through estrogen receptor β (ERβ) [244]. In vitro findings indicate an inhibition of PPARγ transcriptional activity through ERβ, which was in accordance with increased PPARγ activity displayed by ERβ-deficient mice [244]. A rodent model of PPARγ deficiency confirmed a sexually dimorphic response to PPARγ activation by rosiglitazone [245]. The implications of these findings for PPAR-targeted therapy in human diabetic NAFLD require further research.

## 6. Summary

Due to the central role of PPARs in metabolism, the use of PPAR-agonists in T2DM patients offers unique challenges along with opportunities. PPAR-targeted therapies in the field of NAFLD and NASH have demonstrated pleiotropic beneficial effects, both on NAFLD-specific outcomes as well as on a multitude of metabolic functions.

The findings of the NATIVE trial of lanifibranor in particular represent a noteworthy exception in the field of NASH pharmacotherapy, as regression of fibrosis has been demonstrated. These findings, however, need to be further confirmed in the phase 3 NATiV3 trial. While lanifibranor was safe with regard to muscle-related adverse events, reported weight gain of around 3% may hamper use in NAFLD patients with metabolic comorbidities.

While data indicate a possible positive effect on steatosis, no anti-fibrotic effects have been demonstrated for saroglitazar and effects regarding inflammatory activity remain inconclusive. Several clinical trials in NAFLD and NASH are currently ongoing. Overall, saroglitazar has demonstrated a favorable profile regarding metabolic effects and adverse events, although a benefit regarding cardiovascular outcomes remains to be established.

Among the currently available treatment options, pioglitazone is recommended by several NAFLD guidelines. While data on the anti-fibrotic effect in T2DM patients are not fully conclusive, pioglitazone has shown positive effects on NASH inflammatory activity and glucose homeostasis. There has been considerable debate regarding the cardiovascular risk profile of pioglitazone, mainly revolving around the risk of heart failure due to weight gain and fluid retention. The extent to which a positive effect on dyslipidemia translates into overall cardiovascular risk reduction with pioglitazone is therefore unclear.

The role of fibrates both in the treatment of NAFLD and dyslipidemia seems limited. While combination with statins may be safe, fibrates do not offer a relevant benefit regarding cardiovascular outcomes. Results from the PROMINENT trial will offer insights into the cardiovascular benefit of the selective PPARα agonist pemafibrate, where data on histological outcomes in NAFLD are currently lacking. However, since PPARα agonism has been shown to elicit sexually dimorphic effects, both fibrates and pemafibrate for NAFLD should be reviewed with regard to this aspect.

Overall, sexually dimorphic effects of PPARα agonism—and possibly other PPAR isotypes—clearly warrant further exploration. Reporting of trial results stratified by sex might provide further cues and insights into the complex mechanisms of PPAR agonists. Precise phenotyping of trial participants with regard to not only sex but also comorbid conditions, concomitant medications, and lifestyle is needed to adequately capture the multi-systemic effects of PPAR-targeted therapies.

## Figures and Tables

**Table 1:** Evidence from randomized, controlled trials reporting liver-specific outcomes of PPAR-modulating therapy in NAFLD patients, 2016–2021.

Drug Name; Target Pharmaceutical Company	Reference, Country	Study Type; Treatment Duration	Participants	Intervention	Results
Pemafibrate; PPARα Kowa Pharmaceutical	Nakajima et al., 2021 [61], Japan	RCT; 72 weeks	Adults w/NAFLD defined by liver fat content ≥ 10% (MRI-PDFF), liver stiffness ≥ 2.5 kPa (MRE), ALT > 40 U/L for men, >30 U/L for women Exclusion: poorly controlled T2DM (HbA1c ≥ 8%) N = 118 Female: 50 (42%) T2DM: 43 (36%)	Arm (1): Pemafibrate 0.2 mg/day Arm (2): Placebo	Liver-related outcomes ΔIHTG by MRI-PDFF (%): (1): −5.3 (2): −4.2 ΔLSM by MRE (%): (1): −7.3 ^ab^ (2): −1.1 Significant reductions of ALT, γ-GT, and ALP in (1). No significant changes in AST. Metabolic outcomes Significant reduction of TC, LDL-C, non-HDL-C, and TG but also of HDL-C (1).
	Yokote et al., 2021 [62], Japan	Pooled analysis of 6 RCTs; 12 weeks	Adult patients w/hypertriglyceridemia N = 1253 Female: 184 (15%) NAFLD: 534 (43%) T2DM: 449 (36%)	Arm (1): Pemafibrate 0.1 mg/day Arm (2): Pemafibrate 0.2 mg/day Arm (3): Pemafibrate 0.4 mg/day Arm (4): Placebo	Liver-related outcomes Non-significant reductions of AST (−45.5 to −58.1 U/L (1–3) vs. −33.3 U/L (4)). Dose-dependent reductions of ALT, significant for (2) and (3) (−58.5 and −67.2 U/L vs. −18.4 U/L (4)). Significant reductions in γ-GT (−61.1 to −80.6 U/L (1–3) vs. −10.9 U/L (4)). Significant reductions in ALP (1–3); non-significant reductions in bilirubin. Metabolic outcomes Significant reductions of fasting plasma glucose, fasting serum insulin, and HOMA-IR (1–3); no change in HbA1c. Significant reductions of TG and increases of HDL-C (1–3).
Pioglitazone and rosiglitaone; PPARγ	Mantovani et al., 2020 [63], international (USA, Europe, and Asia)	Systematic review of 8 RCTs (pioglitazone 6 and rosiglitazone (2)); 4 to 36 months	Adults w/NAFLD and thiazolidinedione treatment for NAFLD/NASH N = 828 Female: 43% T2DM: 15%	(1): Rosiglitazone 8 mg/day (2): Pioglitazone 30 to 45 mg/day	Liver-related outcomes Significant improvements of liver fat content, NASH, and serum ALT/AST levels (1–2). No significant change in fibrosis stage compared to control in all RCTs, except one (1).
	Musso et al., 2017 [19], international (USA, Europe, and Asia)	Meta-analysis of 8 RCTs (pioglitazone 5 and rosiglitazone (3)); 6 to 24 months	Adults w/NAFLD defined by radiological or histological evidence of steatosis N = 516 (in main analysis of primary outcome; N = 698 participants overall) Female: 333 (48%) T2DM: 142 (20%)	(1): Rosiglitazone 4 to 8 mg/day (2): Pioglitazone 30 to 45 mg/day	Liver-related outcomes Improvement of advanced fibrosis (F3-4 to F0-2; ≥2 stages improvement) in all participants (OR, 95% CI): (1): 1.30, 0.23–7.20 (2): 4.53, 1.52–13.52 Overall: 3.15, 1.25–7.93 Improvement of advanced fibrosis (F3-4 to F0-2; ≥2 stages improvement) in participants with advanced fibrosis (OR, 95% CI): (1): 1.84, 0.29–11.66 (2): 10.17, 2.83–36.54 Overall: 5.84, 2.04–16.71 Improvement of ≥1 fibrosis stage in all participants (OR, 95% CI): (1): 1.18, 0.43–3.25 (2): 4.53, 1.77, 1.15–2.72 Overall: 1.66, 1.12–2.47 NASH resolution in participants with NASH (OR, 95% CI): (1): 2.14, 0.94–4.86 (2): 3.65, 2.32–5.74 Overall: 3.22, 2.17–4.79
Pioglitazone; PPARγ	Bril et al., 2019 [64], USA	RCT; 18 months	Adults w/NASH in liver histology and T2DM Exclusion: T1DM N = 105 Female: 12 (11%)	Arm (1): Pioglitazone 45 mg/day plus vitamin E 400 IU b.i.d. Arm (2) Vitamin E 400 IU b.i.d. Arm (3): Placebo	Liver-related outcomes Reduction in NAS ≥2 points w/o worsening of fibrosis (n%): (1): 54 ^b^ (Δ 35 [14 to 56]) (2): 31 (Δ 12 [−1 to 32]) (3): 19 Reduction of NASH w/o worsening of fibrosis (% of n): (1): 43 ^b^ (Δ 31 [11 to 50]) (2): 33 ^b^ (Δ 21 [2 to 40]) (3): 12 Significant improvements in steatosis, inflammation, and ballooning (1). No significant improvements in fibrosis. Significant reduction in IHTG by 1H-MRS in (1 and 2). Metabolic outcomes Significant improvement in HbA1c (1). Modest increase in HDL-C (1).
	Bril et al., 2019, and Cusi et al., 2018 [65,66], USA	RCT; 18 months	Adults w/NASH on liver histology, and T2DM/prediabetes Exclusion: T1DM, clinically significant renal, pulmonary, or cardiac disease N = 101 Female: 30 (30%) T2DM: 52 (51%)	Arm (1a): Pioglitazone 30 to 45 mg/day in T2DM patients Arm (1b): Pioglitazone 30 to 45 mg/day in prediabetic patients Arms (2a and b): Placebo in T2DM and prediabetic patients	Liver-related outcomes Reduction in NAS ≥2 points (≥2 different categories) w/o worsening of fibrosis (n%): (1 overall, 1a and b): 58 ^b^, 60 ^ab^, and 55 ^a^ (2 overall, 2a and b): 17, 16, and 29 ΔFibrosis stage (SD): (1a and b): −0.5 [0.9] ^b^ and −0.4 [0.9] (2a and b): 0.2 [1.2] and −0.2 [0.7] Significant reduction in IHTG by ^1^H-MRS (1a and b). Metabolic outcomes Increases in hepatic and skeletal muscle insulin sensitivity (1 and b), and in adipose tissue insulin sensitivity (1a). Significant reduction in fasting plasma insulin, and significant increases in adiponectin (1a and b), significant reduction in HbA1c (1a). Improvements in HDL-C and TG (1 and b).
Saroglitazar (EVIDENCES IV); PPARα/γ Zydus Discovery	Gawrieh et al., 2021 [67], USA	RCT; 16 weeks	Adults w/NAFLD based on histology or imaging, ALT ≥ 50 U/L and BMI ≥ 25 kg/m^2^ Exclusion: T1DM, poorly controlled T2DM (HbA1c ≥ 9.0%) N = 106 Female: 49 (46%) T2DM: 56 (53%)	Arm (1): Saroglitazar 1 mg Arm (2): Saroglitazar 2 mg Arm (3): Saroglitazar 4 mg Arm (4): Placebo	Liver-related outcomes Significant dose-dependent ALT reductions (−25.5 to −45.8% (1–3) vs. +3.4% (4)). ALT reduction ≥25% in 64–70% (1–3) of patients compared to 18% (4). ALT reduction ≥50% in 15–52% (1–3) of patients compared to 4% (4). ALT < ULN in 6 patients (3) compared to 0 (4). Significant reductions in AST (−25.4 to 34.9% (1–3) vs. +9.8% (4)), ALP (−17.0 to 35.7% (1–3) vs. +3.3 (4)), and γ-GT (−29.4 to −45.7 (1–3) vs. +10.9 (4)). Significant reduction of steatosis by MRI-PDFF (difference −23.8% (3)) with >30% reduction in 11 patients (3) compared to 2 (4). No significant changes in CK18, LSM, or CAP. Metabolic outcomes Significant improvements in TG (1 and 3), non-significant improvements in HDL-C and LDL-C (3). Significant improvement in HOMA-IR (3), and non-significant improvements in blood glucose, HbA1c, and insulin levels (1 and 3).
Elafibranor (GOLDEN-505); PPARα/δ Genfit	Ratziu et al., 2016 [68], Europe and USA	RCT; 52 weeks	Adults w/NASH on liver histology N = 274 Female: 81 (33%) T2DM: 107 (39%)	Arm (1): Elafibranor 80 mg Arm (2): Elafibranor 120 mg Arm (3): Placebo	Liver-related outcomes Resolution of NASH w/o worsening of fibrosis according to current definition (OR, 95% CI): (1) 2.31, 1.02–5.24 ^b^ (2) 1.48, 0.7–3.14 Overall more pronounced effects in those with severe inflammation (NAS ≥ 4, (1): 3.52, 1.32–9.40) or presence of fibrosis (fibrosis any stage, (1): 3.75, 1.39–10.12). Improvements in γ-GT and ALP (1–2), no changes in ALT. Metabolic outcomes Significant improvements of HbA1, HOMA-IR, and fasting glucose (1) in diabetic patients, as well as improvement in plasma insulin (1–2). Dose-dependent increases in HDL-C (1–2). Decreases in LDL-C and TG (1–2).
Lanifibranor (NATIVE); Pan-PPAR Inventiva Pharma	Francque et al., 2021 [20], international (Europe, Canada, USA, and Australia)	RCT; 24 weeks	Adults w/NASH on liver histology Exclusion: cirrhosis (F4) N = 247 Female: 144 (58%) T2DM: 103 (2%)	Arm (1): Lanifibranor 800 mg Arm (2): Lanifibranor 1200 mg Arm (3): Placebo	Liver-related outcomes Decrease of ≥2 points from SAF activity score w/o worsening of fibrosis (RR, 95% CI): (1) 1.45, 1.00–2.10 ^b^ (2) 1.69, 1.22–2.34 ^b^ Improvement of NASH w/o worsening of fibrosis (RR, 95% CI): (1) 1.70, 1.07–2.71 ^b^ (2) 2.20, 1.49–3.26 ^b^ Improvement of fibrosis ≥1 stage w/o worsening of NASH (RR, 95% CI): (1) 1.15, 0.72–1.85 (2) 1.68, 1.15–2.46 ^b^ Significant reductions in AST, ALT, and γ-GT (1–2). Metabolic outcomes Significant improvements in HOMA-IR (−5.5 to −5.8 (1–2) vs. −1.47 (3)), insulin (−115 to −119 pmol/L (1–2) vs. −36 pmol/L (3)), HbA1c, and fasting glucose (1–2). Significant reduction of TG (1–2), no significant changes in TC, HDL-C, and LDL-C.

^a^ Significant compared to baseline; ^b^ significant compared to placebo; non-significant if not mentioned separately. Abbreviations: Δ, difference; 1H-MRS, proton magnetic resonance spectroscopy; 95% CI, 95% confidence interval; ALP, alkaline phosphatase; ALT, alanine aminotransferase; AST, aspartate aminotransferase; BID, twice daily; BMI, body mass index; CAP, controlled attenuation parameter; CI, confidence interval; EMA, European Medicines Agency; γ-GT, gamma-glutamyltransferase; HbA1c, glycated hemoglobin A1c; HDL-C, high-density lipoprotein cholesterol; HOMA-IR, homeostatic model assessment of insulin resistance; IHTG, intrahepatic triglyceride content; LDL-C, low-density lipoprotein cholesterol; LSM, liver stiffness measurement; MRE, magnetic resonance elastography; MRI-PDFF, magnetic resonance imaging proton density fat fraction; na, not available; NAFLD, non-alcoholic fatty liver disease; NAS, NAFLD Activity Score; NASH, non-alcoholic steatohepatitis; OR, odds ratio; RCT, randomized controlled trial; RR, risk ratio; SAF, steatosis activity fibrosis scoring system; T1DM, type 1 diabetes mellitus; T2DM, type 2 diabetes mellitus; TC, total cholesterol; TG, triglycerides; ULN, upper limit of normal; USA, United States of America; w/, with; w/o, without.

**Table 2:** Ongoing interventional trials of PPAR-targeted therapy in NAFLD/NASH.

Drug Name; Target Pharmaceutical Company	Country	Trial Registration ID Trial Name	Participants (Randomization)	Intervention	Treatment Duration (Weeks)	Primary Outcome(s) Secondary Outcome(s)
Pioglitazone; PPARγ	South Korea	NCT03646292	Adults w/NAFLD (diagnosed on ultrasound and other modalities) and T2DM N = 60 (1:1:1)	Arm (1): Empagliflozin 10 mg Arm (2): Pioglitazone 15 mg Arm (3): Empagliflozin 10 mg + pioglitazone 15 mg	24	Change in hepatic fat content by MRI-PDFF. Change in liver fibrosis by MRE, changes in lipid profiles, liver enzymes, glucose metabolism, and inflammatory biomarkers.
	Pakistan	NCT04976283	Adults w/NAFLD (diagnosed by FibroScan) and T2DM N = 123 (1:1:1)	Arm (1): Pioglitazone 15 mg Arm (2): Empagliflozin 5–12.5 mg Arm (3): Empagliflozin 10 mg + Pioglitazone 15 mg	52	Change in radiologic liver parameters. Changes in liver enzymes, liver fibrosis scores, body weight, body composition, glucose metabolism, and lipid profiles.
	USA	NCT04501406	Adults w/NASH (histologically confirmed) and T2DM N = 138 (1:1)	Arm (1): Pioglitazone 15 mg Arm (2): Placebo	72	Proportion of patients achieving ≥2 points improvement in NAS w/o worsening of fibrosis. Resolution of NASH w/o worsening of fibrosis, improvements in SAF score and NAS, and change in fibrosis.
Saroglitazar; PPARα/γ Zydus Discovery	USA	NCT03639623 EVIDENCES VIII	Adults 6 months post-transplantation for NASH N = 15	Arm (1): Saroglitazar 4 mg	24	Safety (adverse events). Changes in hepatic fat, metabolic flexibility, lipid profiles, liver enzymes, glucose metabolism, pharmacokinetics, and quality of life.
	USA	NCT05011305	Adults w/NASH (histologically confirmed) N = 240 (1:1:1)	Arm (1): Saroglitazar 2 mg Arm (2): Saroglitazar 4 mg Arm (3): Placebo	76	Resolution of NASH w/o worsening of fibrosis. Improvements in fibrosis, NAS and SAF score, changes in lipid profiles, liver enzymes, glucose metabolism, and body weight.
	USA	NCT03617263	Adult females w/NAFLD and PCOSN = 90 (1:1)	Arm (1): Saroglitazar 4 mg Arm (2): Placebo	34	Hepatic fat content by MRI-PDFF. Changes in liver enzymes, liver steatosis, liver fibrosis, BMI, body composition, glucose metabolism, pharmacokinetics, ovarian function, and free androgen index.
	India	NCT04193982	Adults w/NAFLD (histologically confirmed) N = 250 (1:1:1:1)	Arm (1): Saroglitazar 4 mg Arm (2): Vitamin E 400 mg Arm (3): Saroglitazar 4 mg + vitamin E 400 mg Arm (4): Lifestyle intervention	24	Change in NFS. Changes in liver enzymes, lipid profiles, liver fibrosis in histology, NAS, and HbA1c.
Lanifibranor; Pan-PPAR Inventiva Pharma	USA	NCT03459079	Adults w/NAFLD and T2DM N = 44 (1:1)	Arm (1): Lanifibranor 800 mg Arm (2): Placebo	24	Change in IHTG by 1H-MRS. Proportion patients w/ ≥ 30% decrease in IHTG and NAFLD resolution, changes in insulin sensitivity, lipid profiles, glucose metabolism, and biomarkers of fibrosis.
	USA	NCT04849728 NATIV3	Adults w/NASH (histologically confirmed) and fibrosis stages 2–3 N = 2000 (1:1:1)	Arm (1): Lanifibranor 800 mg Arm (2): Lanifibranor 1200 mg Arm (3): Placebo	72	Resolution of NASH and improvement of fibrosis, and time to first clinical outcome event. Resolution of NASH w/o worsening of fibrosis, improvement of fibrosis w/o worsening of NASH, changes in liver enzymes, lipid profiles, glucose metabolism, and quality of life.

Abbreviations: 1H-MRS, proton magnetic resonance spectroscopy; BMI, body mass index; HbA1c, glycated hemoglobin A1c; IHTG, intrahepatic triglyceride content; MRE, magnetic resonance elastography; MRI-PDFF, magnetic resonance imaging proton density fat fraction; NAFLD, non-alcoholic fatty liver disease; NAS, NAFLD Activity Score; NASH, non-alcoholic steatohepatitis; NFS, non-alcoholic fatty liver fibrosis score; PCOS, polycystic ovary syndrome; SAF, steatosis activity fibrosis scoring system; T2DM, type 2 diabetes mellitus; USA, United States of America; w/, with; w/o, without.

**Table 3:** Effects of PPAR-directed therapy on other comorbidities of the metabolic syndrome.

PPAR Target	Drug Name	Overweight and Obesity	Effects on Lipid Levels	Cardiovascular Comorbidities
PPARα	Pemafibrate	No effect	↑ HDL-C ↓(↓) Triglycerides	Unknown; phase 3 trial (PROMINENT) ongoing
	Fibrates	No effect	↑ HDL-C ↓ Triglycerides	Modest decrease of cardiovascular risk in primary and secondary prevention
PPARδ	Seladelpar	No effect	↑ HDL-C ↓ LDL-C ↓ Triglycerides	No data on cardiovascular outcomes; good cardiovascular safety profile
PPARγ	Pioglitazone and other thiazolidinedione	Weight gain (3–7% of body weight)	↑ HDL-C (↑) LDL-C ↓ Triglycerides	Risk reduction for several cardiovascular outcomes; causing fluid retention and edema; possibly increased cardiovascular risk with rosiglitazone
PPARα/γ	Saroglitazar	No effect	(↑) HDL-C (↓) LDL-C ↓ Triglycerides	No data on cardiovascular outcomes; good cardiovascular safety profile
PPARα/δ	Elafibranor	No effect	(↑) HDL-C ↓ Triglycerides	No data on cardiovascular outcomes; good cardiovascular safety profile
Pan-PPAR	Lanifibranor	Mild weight gain (3% of body weight)	↑ HDL-C ↓ Triglycerides	No data on cardiovascular outcomes; good cardiovascular safety profile

Abbreviations: HDL-C, high-density lipoprotein cholesterol; LDL-C, low-density lipoprotein cholesterol; ↑, increases; ↓, lowers; brackets denote conflicting or unclear results.
